# m-EDI Measurement Using Low-Cost Spectrometric Sensors Based on Photodiode Arrays with Narrowband Color Filters: An Exploration of Alternative Calibration Methods

**DOI:** 10.3390/s25237269

**Published:** 2025-11-28

**Authors:** Diego Rodriguez, Javier Ribas, Pablo Quintana-Barcia, David Gacio, Daniel Mallada, Marina S. Perdigao

**Affiliations:** 1 Department of Electrical Engineering, University of Oviedo, 33003 Oviedo, Spainquintanapablo@uniovi.es (P.Q.-B.);; 2 Instituto de Telecomunicações, 3810-193 Aveiro, Portugal; 3 Polytechnic Institute of Coimbra, 3045-093 Coimbra, Portugal

**Keywords:** m-EDI sensor, integrative lighting, circadian illumination, color sensors

## Abstract

Recent studies have highlighted the key role of lighting in regulating circadian rhythms and its impact on human health. Exposure to blue light, especially at specific times of day, is now quantified using the melanopic Equivalent Daylight Illuminance (m-EDI) parameter, defined in the CIE S 026 standard. This parameter is proportional to the integral, in the visible range, of the spectral power distribution and the melanopic sensitivity function, which peaks near 490 nm, and is similar to a Gaussian distribution. Low-cost spectrometric sensors using photodiode arrays and narrowband filters offer a cost-effective way to estimate m-EDI through a weighted sum of channel responses. However, due to inherent sensor variability, individual calibration is recommended. The standard approach involves multiple linear regression to fit the sensor’s output to reference values using a set of test light sources. This method is easy to implement but depends heavily on the selection of calibration illuminants, which must outnumber the channels. This paper analyzes the sensitivity of this method to the sensor’s spectral response and the choice of calibration sources. A revised calibration approach is proposed, selectively discarding channels to reduce deviations from the target response. Applied to several sensors, this method significantly improves calibration accuracy and robustness, reducing the RMS error for several test LEDs from 17.6 to 1.36 lux.

## 1. Introduction

The availability of systems capable of measuring the melanopic equivalent daylight illuminance (m-EDI) at workplaces is essential to ensure lighting conditions that support human health and align with users’ circadian rhythms [1,2,3]. The m-EDI parameter quantifies the circadian stimulation provided by existing lighting, taking into account its effect on the eye’s non-visual photoreceptors, particularly the intrinsically photosensitive retinal ganglion cells (ipRGCs) [3]. This metric is critical for designing environments that promote well-being, alertness, and cognitive performance, as inadequate or poorly calibrated lighting can disrupt sleep-wake cycles, affect mood, and decrease productivity [3,4]. Integrating sensors and control systems that monitor m-EDI enables dynamic adjustment of artificial lighting to complement available natural light, thereby optimizing both visual and biological health while minimizing power consumption. The International Commission on Illumination (CIE) defined the m-EDI parameter in its CIE S 026 standard [1], specifying that its value is proportional to melanopic irradiance, which is calculated as follows:

(1) $$\begin{array}{c} E_{\mathrm{mel}}=\int_{380}^{780}S_{\mathrm{mel}}\left(\lambda \right)\cdot spd\left(\lambda \right)\cdot d\lambda , \end{array}$$

where spd(λ) is the spectral power distribution of the light source, and $S_{\mathrm{mel}}\left(\lambda \right)$ is the normalized melanopic sensitivity function, which peaks at 490 nm in the cyan region of the spectrum. The m-EDI value is equal to 0.754 times $E_{mel}$ [1,4,5].

Recently, based on multiple studies in the field of neurophysiology [2,3] examining the effects of lighting on ipRGCs and their role in melatonin production and suppression mechanisms, a consensus has been established regarding the recommended exposure values to support healthy circadian functioning [4]. This consensus suggests the following:

- m-EDI < 10 melanopic lux for at least three hours before bedtime, to prevent melatonin suppression.
- m-EDI > 250 melanopic lux during the peak daylight hours, to enhance alertness and circadian alignment.

**Figure 1:**
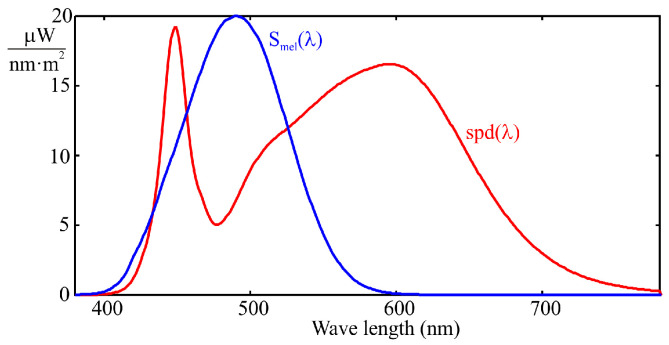
Spectral comparison of a cool white LED (red) and the melanopic sensitivity curve (blue).

However, achieving these spectral levels with conventional white LED lighting, which is typically based on phosphor conversion from primary blue-emitting LEDs, can be challenging due to a relative minimum in the spectral distribution, commonly known as the “cyan gap” (see Figure 1). This gap makes it difficult to meet the condition of m-EDI > 250, as it requires using high lux values, which carries the risk of over-illumination and unnecessary energy consumption. On the other hand, the gap is not large enough to allow compliance with the condition m-EDI < 10 without a significant reduction in lux levels. These limitations have led to the development of new LED technologies that either reinforce or suppress cyan emission, depending on the application.

These technologies allow interior designers to use lighting systems tailored to the intended use of spaces. For example, in rooms intended for use during the hours before sleep, the use of LEDs with low cyan radiation emission, commonly referred to as “zero-blue”, enables maintaining an adequate lux level without the user perceiving an excessively orange-red hue in the light source. Conversely, for spaces where users remain during the central hours of the day, the use of cyan-enhanced LEDs helps minimize the impact on users’ circadian rhythms caused by insufficient exposure to natural sunlight.

There are also adjustable-spectrum LED lamps available on the market, which allow modification of the ratio between photopic and melanopic lux. This enables a single lighting system to comply with the previously mentioned recommendations for m-EDI values, adapting to the time of day and usage conditions.

In this context, having low-cost sensors to measure m-EDI values allows for better optimization of the lighting system in several aspects, including the following:

- In systems with light level or spectrum control, it enables automatic adaptation to existing sunlight, minimizing energy consumption while ensuring compliance with the photopic and melanopic lighting requirements of the application.
- It allows verification that the m-EDI levels users are exposed to meet the recommendations of consensus [4].

As a result of the aforementioned issues, measuring m-EDI in indoor environments, especially workplaces, becomes essential for designing healthy and adaptive lighting solutions. To this end, several sensor technologies are currently available to estimate this parameter [6]:

- Spectrometers: sensors typically based on gratings that disperse incident light onto linear CCD arrays for spectral characterization [7,8,9]. They are generally the most accurate solution but also the most expensive.
- Photodiode arrays with spectral response adjusted by specific color filters: although many RGB sensors are based on this principle [10], currently no commercially available filters adequately emulate the $S_{\mathrm{mel}}\left(\lambda \right)$ sensitivity function.
- Spectrometric sensors with very low resolution: one of the low-cost alternatives currently used in various colorimetric applications is based on the use of low-resolution spectrometric sensors built from photodiode arrays combined with narrowband optical filters (LRS PACF). These sensors include multiple channels, each sensitive to specific regions of the spectrum, which allows for the estimation of several spectral parameters using a single low-cost device. However, they typically require individual calibration to achieve accurate results [11,12,13,14,15].

This paper focuses on the latter devices due to their favorable trade-off between cost, size, and versatility. Specifically, it explores different calibration approaches aimed at accurately estimating m-EDI based on readings from the sensor’s various spectral channels.

Several recent studies describe how the use of sensors of this type can be employed to measure various colorimetric parameters. For example, in [13], the application of a 10-channel LRS-PACF sensor for illuminance measurement is analyzed. The same sensor was used in [16] to measure photosynthetically active radiation. Additionally, the spreadsheets provided by the sensor manufacturer [12] present several alternatives for measuring the chromatic coordinates X, Y, and Z. Besides, several recent studies have addressed the estimation of the m-EDI parameter using low-cost sensors, with approaches that complement the one proposed in this work. Trinh et al. investigated the use of RGB sensors to estimate circadian stimulus, developing processing algorithms that enable a reasonably accurate approximation of m-EDI [10]. Moreover, Mohammadian et al. introduced a portable IoT-based device built on photodiodes for continuous m-EDI monitoring in circadian health applications, validating its performance against reference-grade spectrometers [17]. Although these studies rely on different sensor architectures, they share the common goal of providing accessible and efficient solutions for m-EDI measurement, thereby reinforcing the relevance of the LRS-PACF sensor-based approach developed in this article.

**Figure 2:**
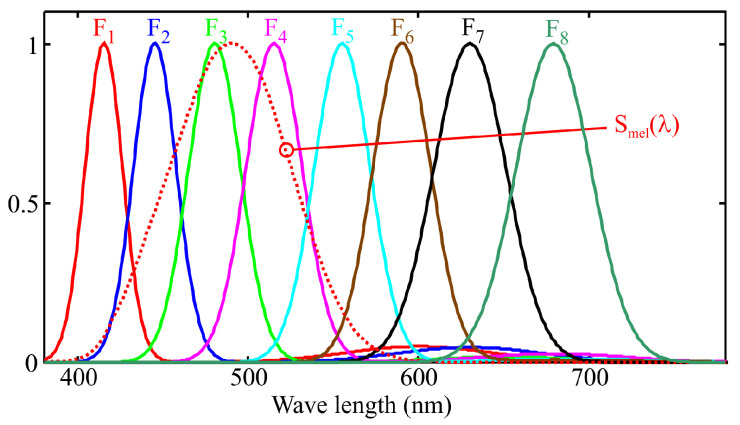
Example of the response curves of a simulated 8-channel sensor.

To illustrate how an LRS-PACF sensor can be used to obtain the spectral response required to measure m-EDI, Figure 2 shows the normalized spectral response of a representative eight-channel sensor with characteristics comparable to those of certain commercially available models. Each channel, denoted as $F_{i}\left(\lambda \right)$, is sensitive to a specific region of the visible spectrum. The spectral response of currently available commercial sensors varies between manufacturers and models. Although the methodology proposed in this work is applicable to different types of responses, the graphs shown below correspond to sensors whose channels exhibit responses similar to a Gaussian bell curve. This choice is due to the fact that this is the most common type of response in LRS-PACF sensors.

The output of each sensor’s channel ($ch_{1}$ to $ch_{8}$) is proportional to the integral, over the visible spectrum, of the product of the light source’s spectral power distribution at the measurement point, spd(λ), and the corresponding channel sensitivity curve (F_1_ to F_8_ in Figure 2). Mathematically, this can be expressed as:

(2) $$\begin{array}{c} ch_{j}=\int_{380}^{780}F_{j}\left(\lambda \right)\cdot spd\left(\lambda \right)\cdot d\lambda . \end{array}$$

**Figure 3:**
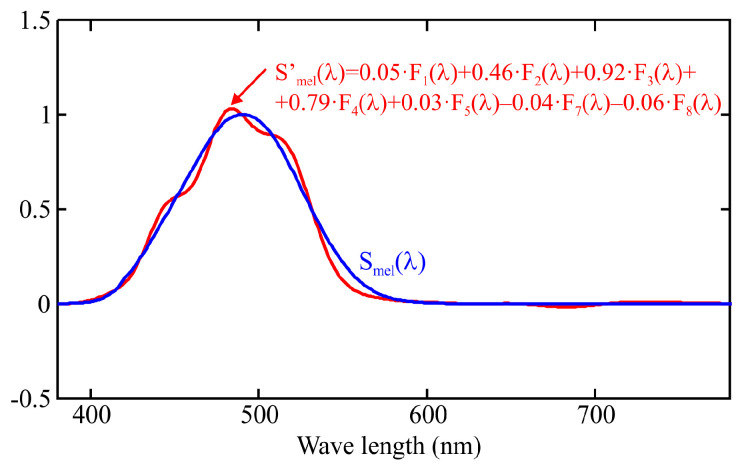
Approximation of the melanopic sensitivity curve as a weighted sum of the responses from the eight channels of the example sensor.

Melanopic irradiance, $E_{\mathrm{mel}}^{\prime }$, is computed as a weighted linear combination of the channel outputs:

(3) $$\begin{array}{c} E_{\mathrm{mel}}^{\prime }=\sum_{j=1}^{8}ch_{j}\cdot \beta_{j}, \end{array}$$

where $\beta_{j}$ is the weighting coefficient obtained from calibration. This is equivalent to approximating the $S_{\mathrm{mel}}\left(\lambda \right)$ curve as a combination of the spectral responses of the sensor channels, as shown in Figure 3, according to the following expression:

(4) $$\begin{array}{c} S_{\mathrm{mel}}^{\prime }=\sum_{j=1}^{8}\beta_{j}\cdot F_{j}\left(\lambda \right). \end{array}$$

The error of this approximation depends on the number of sensor channels, the spectral response of each channel, and the calibration method used to determine the weighting coefficients. The calibration procedure commonly recommended by manufacturers, based on multivariable linear regression (MLR), is further analyzed in Section 3 showing that it may lead to significant deviations between the estimated spectral response, $S_{\mathrm{mel}}^{\prime }\left(\lambda \right)$, and the theoretical one, $S_{\mathrm{mel}}\left(\lambda \right)$, for certain wavelengths [18].

This work is structured into several sections that progressively address the development and validation of a spectral calibration system for multichannel sensors. Section 2 describes the experimental procedure used to characterize the spectral response of each sensor channel, using a monochromatic sweep as the reference technique [18]. Section 3 introduces multiple linear regression (MLR) as the calibration method, outlining both the theoretical foundations and the practical implementation of the model. Section 4 and Section 5 present the application of the proposed method to two commercial sensors with 10 and 13 channels, respectively: the AS7341 and AS7343 models from ams OSRAM [11,12]. These sections include experimental results and analyze sensor performance under various test conditions. Section 6 proposes a fitting refinement strategy based on Selective Channel Elimination (SCE), which improves accuracy by discarding channels that introduce significant deviations from the target sensitivity curve after applying MLR. Finally, Section 7 provides a quantitative comparison between the outcomes of the MLR model and the SCE-enhanced approach, using a range of experimental tests involving multiple sensors and illuminants. The conclusions summarize the key findings and offer practical recommendations for implementing LRS-PACF sensors in circadian lighting applications.

## 2. Method for Obtaining the Spectral Response of Each Sensor Channel

As described in the previous section, the spectral response of each channel in an LRS-PACF can be used to graphically represent the resulting spectral fit obtained by applying a weighted linear combination of the channel outputs to estimate the m-EDI parameter, as shown in Figure 3. The following sections further discuss how this representation enables the evaluation of the performance of different methods used to compute the weighting coefficients.

This section describes the procedure used to characterize the sensor’s spectral response using a monochromatic light sweep covering its entire sensitivity range. In some sensors, the sensitivity extends beyond the visible spectrum because certain channels exhibit cross-sensitivity to infrared (IR) or ultraviolet (UV) wavelengths, as reported in [19] and detailed in Section 4 and Section 5. The absence of response data in these spectral regions can lead to inaccurate or non-representative results when evaluating light sources that emit significant IR or UV radiation, such as incandescent lamps or natural sunlight.

**Figure 4:**
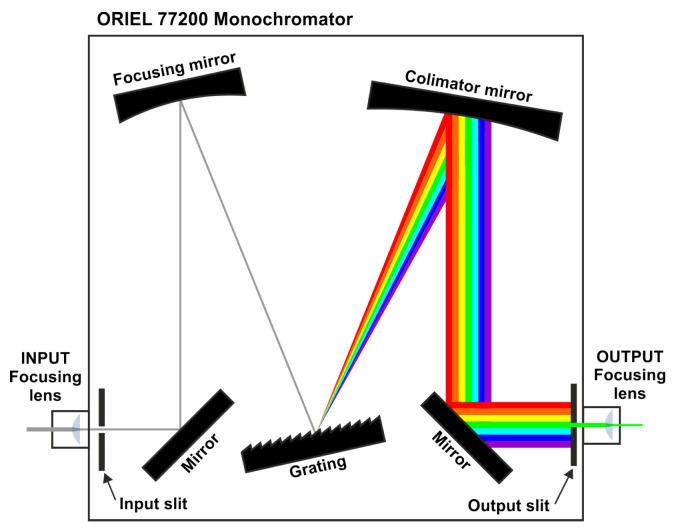
Operation of the tunable monochromatic light source used.

The core component of the spectral characterization system is a tunable monochromatic light source built from a continuous-spectrum halogen lamp coupled with a monochromator (see Figure 4) [15,20]. The monochromator separates the light into its spectral components using a movable diffraction grating. An adjustable slit controls the bandwidth of the output spectrum: the narrower the slit, the more monochromatic the output. By varying the relative angle between the grating and the incident light beam, the central wavelength of the resulting spectrum can be tuned. A key limitation of this system is that, as the output becomes more monochromatic, the transmitted optical power decreases significantly. Consequently, the signal reaching the sensor may fall close to its noise floor, hindering accurate response measurements for certain channels. To alleviate this effect, collimating lenses are placed at both the input and output of the monochromator to optimize the use of the available optical power. The monochromator used was the Oriel (US) model 77200, equipped with a Newport 53-330R grating. To compensate for the low reflectivity of this grating in the infrared region, a Thorlabs OSL2 halogen lamp was employed, featuring a reflector optimized to maximize infrared output.

**Figure 5:**
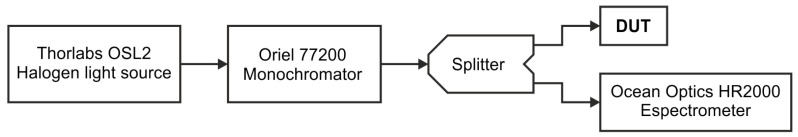
Diagram of the system used for spectral characterization of the sensors.

The complete setup used for the spectral characterization of the sensors is illustrated in Figure 5. The output from the previously described monochromator is routed through an optical fiber splitter, delivering equivalent signals to both the Device Under Test (DUT) and an Ocean Optics HR2000+ spectrometer, which serves as the reference sensor during the calibration process. To minimize transmission losses, a high-performance holographic diffuser (model #35-869, Edmund Optics) was employed as cosine correction optics for the DUT. Due to its relatively high cost compared to the sensor itself, this diffuser was not intended for use in the final application. Since the spectral characterization of the sensor is performed using filters different from those employed in the final application, the response may vary at certain wavelengths. However, the complete spectral response obtained using the setup shown in Figure 5 is used solely to illustrate the graphical impact of applying different calibration procedures. For the final sensor calibration, the low-cost diffusers intended for the target application were used.

The ideal scenario for obtaining the spectral response of the sensor would involve a highly monochromatic output from the monochromator, delivering full-width at half maximum (FWHM) values below 2 nm and sufficient intensity to ensure meaningful readings from the sensor channels across the entire wavelength range of interest. In such conditions, the spectral response could be directly estimated from the sensor readings at each wavelength. However, with the characterization setup used (see Figure 5), it was not possible to meet these requirements. Therefore, a trade-off had to be established between the halogen lamp power and the aperture of the input and output slits, aiming to ensure an adequate signal level in the channels of the DUT while avoiding saturation of the reference spectrometer. As a result, the spectrum applied to the sensors exhibited Full Width at Half Maximum (FWHM) values ranging from 12 to 17 nm, depending on the wavelength, and thus could not be considered strictly monochromatic for the purpose of calculating the sensor’s spectral response. To compensate for this effect, it was necessary to record both the full spectrum of the monochromator output and the corresponding readings from each DUT channel for every evaluated wavelength.

**Figure 6:**
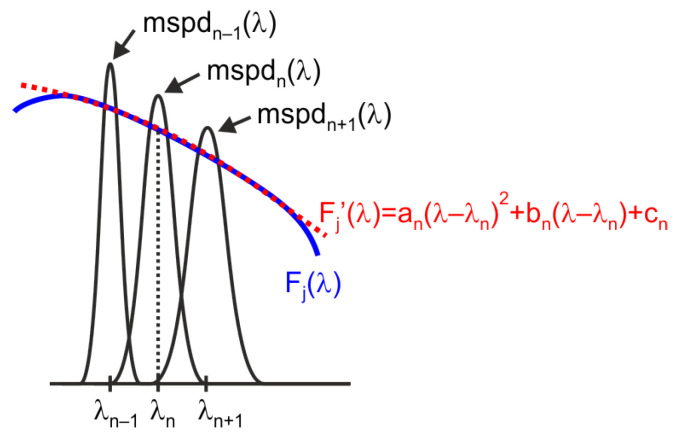
Method used to compensate for the variation in the monochromatic spectra employed for sensor characterization.

Figure 6 illustrates the procedure used to compensate for spectral shifts in the quasi-monochromatic signals employed for characterization. This procedure is based on approximating the actual response of the sensor’s channel under characterization, $F_{j}\left(\lambda \right)$, around each considered wavelength, $\lambda_{n}$, using a polynomial series expansion [21,22]. The best results are obtained using a second-order polynomial approximation, $F_{j}^{\prime }\left(\lambda \right)$, of the sensor channel characteristic.

Assuming that the polynomial series approximation is valid in the vicinity of the considered wavelength $\lambda_{n}$, which includes the wavelengths with significant values in the quasi-monochromatic spectra used for the characterization, denoted as $mspd_{n}\left(\lambda \right)$, as well as the adjacent spectra $mspd_{n-1}\left(\lambda \right)$ and $mspd_{n+1}\left(\lambda \right)$, the sensor readings for channel *j* at samples n−1, *n*, and n+1 can be approximated by the following expressions:

(5) $$\begin{array}{c} ch_{j,n-1}≅\int_{380}^{780}mspd_{n-1}\left(\lambda \right)\cdot \left(a_{n}{\left(\lambda -\lambda_{n}\right)}^{2}+b_{n}\left(\lambda -\lambda_{n}\right)+c_{n}\right)\cdot d\lambda , \end{array}$$

(6) $$\begin{array}{c} ch_{j,n}≅\int_{380}^{780}mspd_{n}\left(\lambda \right)\cdot \left(a_{n}{\left(\lambda -\lambda_{n}\right)}^{2}+b_{n}\left(\lambda -\lambda_{n}\right)+c_{n}\right)\cdot d\lambda , \end{array}$$

(7) $$\begin{array}{c} ch_{j,n+1}≅\int_{380}^{780}mspd_{n+1}\left(\lambda \right)\cdot \left(a_{n}{\left(\lambda -\lambda_{n}\right)}^{2}+b_{n}\left(\lambda -\lambda_{n}\right)+c_{n}\right)\cdot d\lambda . \end{array}$$

In this way, for each wavelength $\lambda_{n}$, a system of three equations with three unknowns is obtained, which allows the determination of the coefficients of the polynomial used to approximate the sensor’s response. The estimated response of channel j at wavelength $\lambda_{n}$ corresponds to:

(8) $$\begin{array}{c} F_{j}\left(\lambda_{n}\right)≅c_{n}. \end{array}$$

As previously mentioned, the proposed approach requires that the sensor’s response can be approximated by a second-order polynomial within the wavelength range corresponding to three consecutive measurements. For instance, if the monochromator output spectrum has a full width at half maximum (FWHM) of 12 nm and measurements are taken at 2 nm intervals, the approximation will yield acceptable results provided that the sensor channel responses can be accurately modeled using second-order polynomials over 16 nm intervals.

## 3. Adjustment Using the Multivariable Linear Regression Procedure

As mentioned in Section 2, one of the possible methods for calibrating sensors used in m-EDI measurements with low-resolution spectrometers based on LRS-PACF relies on MLR [13,17,23]. For example, this is the calibration method recommended by ams OSRAM (DE) for AS7341 and AS7343 used in Section 4 and Section 5 of this study.

The use of this method does not require the detailed spectral response of the sensor. It is sufficient to compare the readings of each channel with the reference sensor using a number of illuminants greater than the number of channels available in the sensor under calibration [11,12]. The coefficients obtained with this method are those that minimize the measurement error for these illuminants and their linear combinations.

### 3.1. Procedure for Multivariable Linear Regression

The application of the MLR method for zero-intercept functions is based on finding a relationship of the form:

(9) $$\begin{array}{c} Y=\beta_{1}\cdot X_{1}+\beta_{2}\cdot X_{2}+\ldots +\beta_{n}\cdot X_{n}+\varepsilon , \end{array}$$

where the response variable, *Y*, is related to the predictor variables $X_{1}$, $X_{2}$, …, $X_{n}$, whereas the regression coefficients $\beta_{1}$, $\beta_{2}$, …, $\beta_{n}$ are computed to minimize the error term ε over the available sample data. For this dataset, the following condition holds:

(10) $$\begin{array}{c} y_{i}=\sum_{j=1}^{n}\beta_{j}\cdot x_{i,j}+\varepsilon_{i}\;for:i\in \left\{1,2,\ldots ,m\right\}, \end{array}$$

where *m* is the number of samples. The quadratic error function, *E*, is given by:

(11) $$\begin{array}{c} E\left(\beta_{1},\beta_{2},\ldots ,\beta_{n}\right)=\sum_{i=1}^{m}\varepsilon_{i}^{2}=\sum_{i=1}^{m}{\left(y_{i}-\sum_{j=1}^{n}\beta_{j}\cdot x_{i,j}\right)}^{2}. \end{array}$$

In order to minimize the quadratic error, the least squares estimators, denoted by ${\hat{\beta }}_{1}$, ${\hat{\beta }}_{2}$, …, ${\hat{\beta }}_{n}$, must satisfy:

(12) $$\begin{array}{c} {\left.\frac{\partial E}{\partial \beta_{j}}\right|}_{\left({\hat{\beta }}_{1},{\hat{\beta }}_{2},\ldots ,{\hat{\beta }}_{n}\right)}=0. \end{array}$$

In matrix form, the quadratic error function can be expressed as:

(13) $$\begin{array}{c} E\left(\beta \right)=\sum_{i=1}^{m}\varepsilon_{i}^{2}={\left(Y-X\cdot \beta \right)}^{T}\cdot \left(Y-X\cdot \beta \right). \end{array}$$

Applying condition (12) to (13) yields:

(14) $$\begin{array}{c} X^{T}\cdot X\cdot \hat{\beta }=X^{T}\cdot Y. \end{array}$$

Therefore, the estimator obtained by the method of least squares is given by:

(15) $$\begin{array}{c} \hat{\beta }={\left(X^{T}\cdot X\right)}^{-1}\cdot X^{T}\cdot Y. \end{array}$$

In order for the inverse matrix of the term $\left(X^{T}\cdot X\right)$ to exist, the number of illuminants used must be greater than or equal to the number of channels of the sensor to be calibrated.

**Figure 7:**
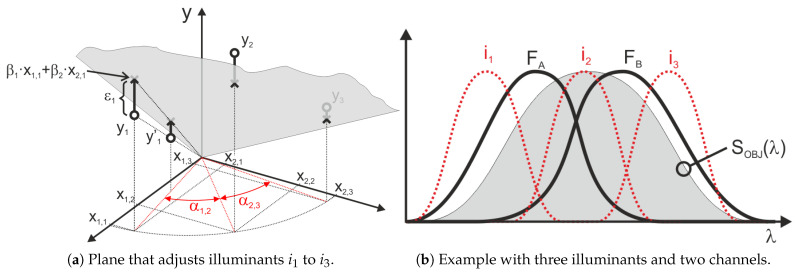
Two-channel graphical representation of the MLR method.

As an example, Figure 7 shows a graphical representation of the approximation provided by the MLR method for a particular case of a sensor with only two channels ($x_{1}$ and $x_{2}$). As previously mention, with only two channels, it is necessary to use at least two illuminants. In this example, three illuminants (i_1_ to i_3_) have been used, so the MLR method allows calculating the plane that minimizes the error for the points defining the aforementioned illuminants. Figure 7a shows in gray the plane that would approximate the measurements corresponding to illuminants i_1_, i_2_, and i_3_ of Figure 7b. Illuminant i_1_ produces a significantly higher output in channel F_A_ than in channel F_B_, which places it closer to the left axis in Figure 7a. Illuminant i_2_ affects both channels approximately equally, resulting in a response that is roughly equidistant from both horizontal axes.

The MLR method minimizes the absolute error; therefore, the point denoted by $y_{1}^{\prime }$ in Figure 7 would carry less weight in the error calculation than point $y_{1}$, even though both correspond to the same relative error.

The major drawback of the MLR method is its sensitivity to multicollinearity, where highly correlated predictors lead to unstable coefficient estimates and poor interpretability. This effect can be illustrated using the example in Figure 7. Low values of angles $\alpha_{1,2}$ and $\alpha_{2,3}$ increase the correlation between predictors, causing the estimated coefficients $\beta_{1}$ and $\beta_{2}$ to become unstable due to the strong sensitivity of this adjustment to errors in the input data. By observing Figure 7b, it can be inferred that the angles $\alpha_{1,2}$ and $\alpha_{2,3}$ decrease when the areas obtained from integrating the curves, calculated as the product of the illuminant spectra i_1_, i_2_, and i_3_ with the sensitivity functions F_1_ and F_2_, become closer. In the example shown in Figure 7b, this effect may occur either when the peaks of i_1_ and i_3_ shift closer to i_2_, or when these illuminants are less monochromatic and exhibit larger FWHM values. An interesting special case occurs when the target curve is constant and equal to zero, meaning the optimal fit would coincide with the plane y=0, with both $\beta_{1}$ and $\beta_{2}$ equal to zero. In this scenario, small values of $\alpha_{1,2}$ and $\alpha_{2,3}$ lead to highly unstable estimates for $\beta_{1}$ and $\beta_{2}$, typically with opposite signs and far from zero. This phenomenon is referred to as the “pendulum effect” in later sections of this article. In the scientific literature, numerous alternative multivariable regression techniques have been proposed to address the limitations of standard MLR, particularly those arising from multicollinearity. Among these approaches are channel weighting regularization, variable selection methods, and ridge regression [24,25]. These techniques introduce distinct strategies aimed at stabilizing coefficient estimates and mitigating the disproportionate influence of specific channels on model behavior. In Section 6.3 of this article, authors present an alternative method that exploits the fact that multicollinearity predominantly occurs between adjacent channels when monochromatic illuminants are applied to an LRS-PACF sensor, whose spectral response resembles that depicted in Figure 2.

### 3.2. Application of the Multivariable Linear Regression Procedure for m-EDI Calculation

The procedure described in the previous section can be used to determine the regression coefficients for estimating the m-EDI value using an LRS-PACF sensor. When using `m’ illuminants to characterize an *n*-channel sensor, the readings from each channel can be expressed in matrix form as follows:

(16) $$X_{i,j}=ch_{i,j}\;for:i=1,\ldots ,m;j=1,\ldots ,n,$$

where ch_i,j_ denotes the reading of channel ‘j’ under illuminant ‘i’. The m-EDI values obtained from the reference sensor for each illuminant can be expressed in matrix form as follows:

(17) $$RefM_{i}={m-\mathrm{EDI}}_{i}\;for:i=1,\ldots ,m.$$

The weighting coefficients β that minimize the quadratic error can be obtained using the following expression:

(18) $$\beta ={\left(X^{T}\cdot X\right)}^{-1}\cdot \left(X^{T}\cdot RefM\right).$$

For any given illuminant, the m-EDI value estimated by the sensor corresponds to:

(19) $$m-\mathrm{EDI}≅\sum_{j=1}^{n}\beta_{j}\cdot ch_{j}.$$

This procedure can also be used to obtain the calibration coefficients for measuring other colorimetric parameters that are calculated in a similar way, that is, parameters proportional to the integral of the product of the spectral distribution and a sensitivity curve, such as lux, tristimulus X-Y-Z values, and other α-opics.

In the following sections, two sensors with 10 and 13 channels will be analyzed and compared. It will be shown that the main drawback of the MLR procedure described in this section lies in its susceptibility to the spectrum employed in the illuminants or to the spectral response of the sensor channels, which may lead to multicollinearity and provide estimations that deviate significantly from the true values.

## 4. Example of a 10-Channel Sensor Calibration with Multivariable Linear Regression Procedure

**Figure 8:**
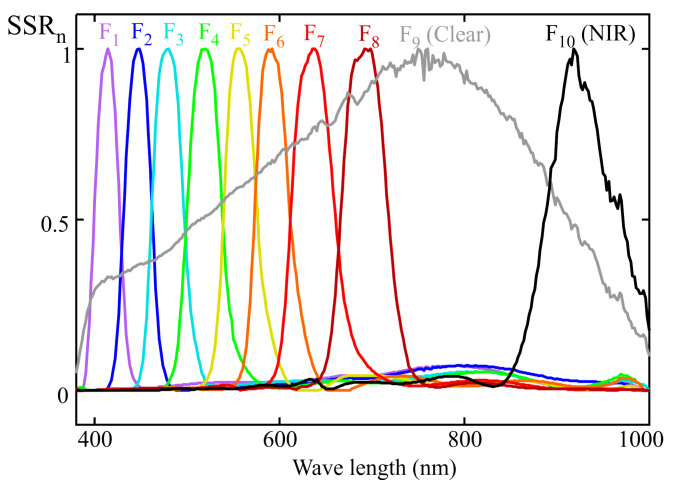
Experimental normalized spectral sensitivity response of a AMS-OSRAM (DE) AS7341 sensor.

Figure 8 shows the normalized spectral sensitivity response (SSRn) of a 10-channel color sensor (model AS7341) from AMS-OSRAM (DE), experimentally obtained using the procedure described in Section 2. This sensor includes eight channels (from F_1_ to F_8_) sensitive to specific regions of the visible spectrum, one channel covering the entire visible range (F_9_, CLEAR), one channel for near-infrared (NIR) measurement (F_10_), and an additional channel for flicker measurement. The last two channels were not used in this study. Figure 8 also reveals cross-sensitivity between channels, such that, for instance, the channels measuring the violet-blue region exhibit non-zero gain at wavelengths close to 800 nm [11].

The aim of this section is to use the full spectral response of the sensor shown in Figure 8 to calculate the spectral response resulting from applying the MLR method with a reduced set of illuminants. This allows for a comparison between the target and the calibrated spectral responses. In this case, the target spectral response corresponds to the melanopic sensitivity curve, $S_{\mathrm{mel}}$.

**Figure 9:**
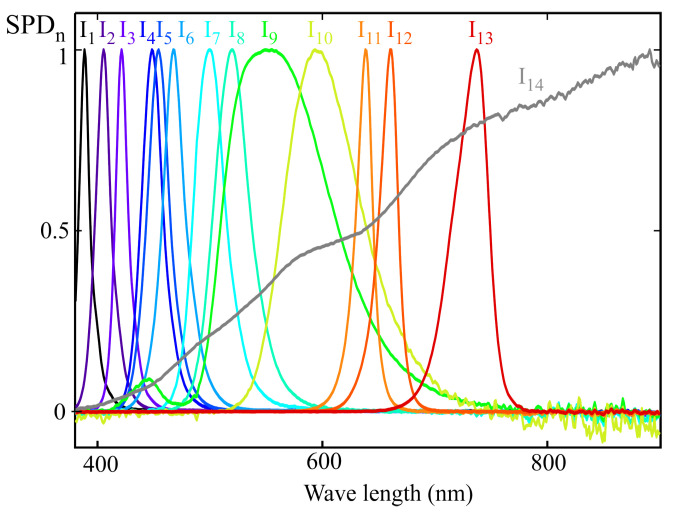
Normalized spectral power distribution of the reference illuminants used for sensor calibration.

Figure 9 shows the normalized spectral power distributions of the 14 light sources used for sensor calibration, following the procedure described in Section 3. These illuminants were carefully selected to ensure full coverage of the visible spectrum while avoiding linear dependence among their spectral profiles. Care was also taken to ensure that no wavelength within the visible range was left without a significant contribution from at least one of the sources. The main specifications of these illuminants are summarized in Table 1.

To apply the MLR procedure, the readings of each sensor channel were estimated using the spectral response (Figure 8) and the SPD of each illuminant (Figure 9). In the general case where the full spectral response from the sensor is not available, the direct readings of each channel would be used to construct the matrix *X* in (18). The m-EDI value corresponding to the *RefM* term in this equation was calculated from the illuminants’ SPD.

**Figure 10:**
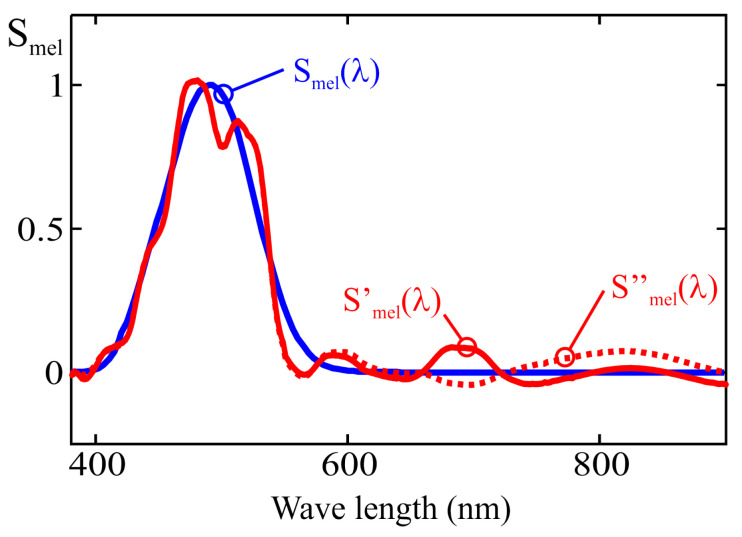
Real and approximated melanopic sensitivity curves using the MLR method for two sets of illuminants.

Figure 10 shows a comparison between the melanopic sensitivity curve, $S_{\mathrm{mel}}\left(\lambda \right)$ and the fit obtained from the MLR calibration process under two conditions:

- Using all the illuminants in Figure 9: the $S_{\mathrm{mel}}^{\prime }\left(\lambda \right)$ curve;
- Using all the illuminants in Figure 9 except the one corresponding to a halogen lamp ($I_{14}$): the $S_{\mathrm{mel}}^{\prime \prime }\left(\lambda \right)$.

In this second case, after removing the illuminant corresponding to a halogen lamp, the reduced energy contribution in the near-infrared region from the remaining light sources results in a poorer spectral fit above 750 nm. As shown in Figure 8, channels F_1_, F_2_, and F_3_ exhibit some sensitivity to wavelengths between 700 and 900 nm. If the calibration illuminants do not emit energy within this range, the fitting error in that part of the spectrum is not considered when applying the MLR method, which can lead to deviations such as the one observed in Figure 10. This highlights the importance of ensuring that the illuminants used for calibration provide sufficient energy across all wavelengths to which the sensor is sensitive. It is also noted that the spectral curves shown in Figure 10 were obtained without using channel F_10_ (NIR).

## 5. Example of a 13-Channel Sensor Calibration with Multivariable Linear Regression Procedure

From the example in the previous section, it can be inferred that the spectral response obtained with an LRS-PACF sensor using the MLR calibration depends on the spectral response of the sensor’s channels and the illuminants used. Employing a sensor with a higher number of channels does not always result in a more accurate spectral response fit, particularly when this increase exacerbates multicollinearity among sensor readings. To illustrate this point, this section analyzes the fit obtained by MLR calibration for a 13-channel sensor.

**Figure 11:**
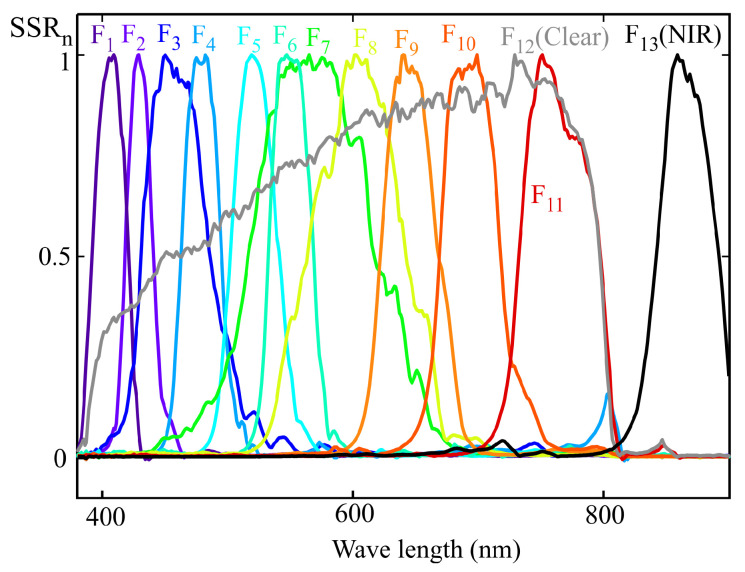
Experimental normalized spectral sensitivity response (SSRn) of a AS7343 sensor.

Figure 11 shows the normalized spectral response of an ams OSRAM sensor (model AS7343), obtained using the procedure described in Section 2. This sensor features 11 channels sensitive to different regions of the visible spectrum (F_1_ to F_11_), one channel sensitive across the entire visible spectrum (F_12_, CLEAR), and one channel sensitive in the NIR region (F_13_, NIR). The latter channel was not used in this study.

The same group of illuminants used in the previous section was employed for calibration; their normalized SPDs can be seen in Figure 9. As in the previous example, the MLR calibration procedure was applied to obtain the weighting coefficients that allow the estimation of melanopic irradiance. The m-EDI value is proportional to this measurement. Also, as in the previous example, the sensor channel readings were estimated from the illuminants’ SPD and the spectral response shown in Figure 11, rather than using direct readings.

The weighting coefficients obtained from the normalized characteristics of channels F_1_ to F_12_ using the MLR procedure, correspond to (20):

(20) $$\begin{array}{c} S_{\mathrm{mel}}^{\prime }\left(\lambda \right)=-0.01\cdot F_{1}\left(\lambda \right)+0.09\cdot F_{2}\left(\lambda \right)+0.01\cdot F_{3}\left(\lambda \right)\\ \;-0.28\cdot F_{4}\left(\lambda \right)-4.2\cdot F_{5}\left(\lambda \right)+9.88\cdot F_{6}\left(\lambda \right)\\ \;-7.27\cdot F_{7}\left(\lambda \right)-8.79\cdot F_{8}\left(\lambda \right)+2.23\cdot F_{9}\left(\lambda \right)\\ \;-2.77\cdot F_{10}\left(\lambda \right)+1.33\cdot F_{11}\left(\lambda \right)+0.42\cdot F_{12}\left(\lambda \right). \end{array}$$

The coefficients in this expression are those that minimize the squared error in the estimation of melanopic illuminance for the calibration illuminants shown in Figure 9. In this expression, it can be observed that the sensor channels with peak responses at wavelengths close to the maximum of the melanopic sensitivity curve (channels F_3_, F_4_, and F_5_) do not exhibit the highest assigned weights, as would be expected. It can also be seen that some coefficients take strongly negative values, such as the one associated with channel F_8_.

**Figure 12:**
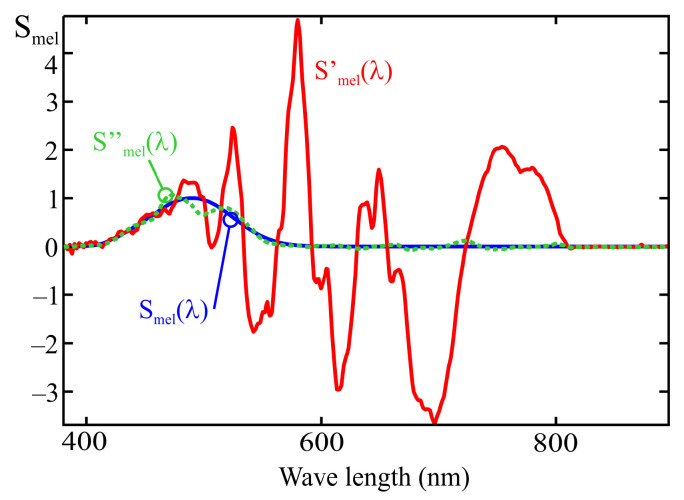
Real melanopic sensitivity curves [($S_{\mathrm{mel}}\left(\lambda \right)$], approximated using the MLR method for the AS7343 sensor [$S_{\mathrm{mel}}^{\prime }\left(\lambda \right)$], and optimized using the full spectral response of the sensor $S_{\mathrm{mel}}^{\prime \prime }\left(\lambda \right)$.

The graphical representation of the spectral sensitivity obtained from this equation corresponds to the $S_{\mathrm{mel}}^{\prime }\left(\lambda \right)$ trace in Figure 12. As can be seen, the deviation of the resulting characteristic from the target curve is considerable at certain wavelengths, which indicates strong multicollinearity among the sensor readings.

Since the full spectral response of the sensor is available, it is possible to determine the optimal weighting coefficients that minimize the deviation over the entire spectral range considered (380–900 nm, evaluated at 1 nm intervals). The resulting expression is as follows:

(21) $$\begin{array}{c} S_{\mathrm{mel}}^{\prime \prime }\left(\lambda \right)=\;-0.08\cdot F_{1}\left(\lambda \right)+0.35\cdot F_{3}\left(\lambda \right)+0.65\cdot F_{4}\left(\lambda \right)+0.64\cdot F_{5}\left(\lambda \right)\\ \;-0.14\cdot F_{6}\left(\lambda \right)-0.01\cdot F_{7}\left(\lambda \right)-0.11\cdot F_{8}\left(\lambda \right)-0.16\cdot F_{9}\left(\lambda \right)\;\\ -0.29\cdot F_{10}\left(\lambda \right)-0.32\cdot F_{11}\left(\lambda \right)+0.28\cdot F_{12}\left(\lambda \right). \end{array}$$

This expression corresponds to the dashed line $S_{\mathrm{mel}}^{\prime \prime }\left(\lambda \right)$ shown in Figure 12. As can be observed, the adjustment can be significantly improved when the full spectral response of the sensor is known, at the expense of a considerable increase in the complexity of the characterization process. As an example, for the sensor’s spectral characterization, a sweep from 360 nm to 900 nm at 2 nm intervals was performed, resulting in 270 measurements instead of the 14 required to derive the model in (20) using the MLR method. Since in both cases the acquisition of these measurements represents the most time-consuming stage of the calibration process, calibration using the MLR method is approximately 19 times faster than a full spectral characterization. However, the MLR method minimizes the squared error for the calibration illuminants. Although the deviation from the target response $S_{\mathrm{mel}}\left(\lambda \right)$ of the spectral characteristic corresponding to the curve $S_{\mathrm{mel}}^{\prime }\left(\lambda \right)$ is much greater than that of $S_{\mathrm{mel}}^{\prime \prime }\left(\lambda \right)$, the mean squared error calculated over the calibration illuminants is higher for the latter.

## 6. Improvements Proposed in the Fitting and Calibration Procedure

As discussed in previous sections, the MLR procedure for calibrating low-resolution spectrometric sensors requires simpler equipment and significantly fewer measurements than those needed to determine the spectral response of each sensor channel using a monochromatic light sweep. However, when the spectral response obtained through the MLR procedure is compared to the target curve ($S_{\mathrm{mel}}\left(\lambda \right)$ in the previous examples), noticeable deviation may appear depending on the behavior of the sensor being calibrated and the illuminants used.

Although the MLR procedure yields minimal error for the illuminants used during calibration, the weighting coefficients obtained through this method become inadequate when applied to spectra that differ from any linear combination of those illuminants, potentially resulting in unacceptable measurement errors. For example, the fit shown in Figure 12 of the previous section leads to an error of 30.8% when a CIE D65 standard illuminant is used, and increases to 49.6% for the CIE LED B_3_ standard illuminant. The deviation becomes even more critical when measuring highly discontinuous spectra, such as those produced by gas discharge lamps (for instance, up to 325% error for the CIE F_11_ standard illuminant), resulting in substantial inaccuracies when estimating the m-EDI parameter for many commonly used light sources.

When comparing the sensor response with the target sensitivity curve, $S_{\mathrm{mel}}\left(\lambda \right)$, an accurate fit across the entire spectral range requires that the main channels, i.e., those whose peak sensitivity is closest to the maximum of the target curve (F_3_, F_4_, and F_5_), be assigned higher weighting coefficients than the others. This behavior results from the fact that the secondary channels primarily serve to compensate for the cross-sensitivity of the main ones. The deviation of the spectral response, $S_{\mathrm{mel}}^{\prime }\left(\lambda \right)$, from the reference melanopic sensitivity curve (see Figure 12), is largely caused by the values assigned to the weighting coefficients of the secondary channels in (20). For instance, the value 9.88 associated with the normalized reading of channel F_6_ is greater in absolute value than that of the weighting coefficient of channel F_5_ (−4.2), resulting in a pendulum effect and a noticeable deviation in response at 757 nm (see Figure 9).

In an idealized scenario where the sensor channels behave perfectly, meaning they have bell-shaped response curves resembling narrow Gaussian distributions compared to the target sensor response ($S_{\mathrm{mel}}\left(\lambda \right)$ in this article), with peak sensitivities approximately evenly spaced across the relevant wavelength range and negligible sensitivity outside their main lobe, all weighting coefficients would be positive to minimize deviation from the desired response.

In practice, moderately negative values in the weighting coefficients may help mitigate the effects of cross-sensitivity between channels. For instance, a slightly negative coefficient applied to channel F_11_ may partially compensate for the 800 nm response featured by channel F_4_ in the example discussed in the previous section (see Figure 11). Nevertheless, it is undesirable for such a negative coefficient to have an absolute value comparable to those of the channels whose response lies closer to the peak of the target curve, $S_{\mathrm{mel}}\left(\lambda \right)$, specifically F_3_, F_4_, and F_5_, as this could lead to significant deviations such as those observed in the curve for $S_{\mathrm{mel}}^{\prime }\left(\lambda \right)$ in Figure 12.

This fitting can be improved using two complementary strategies: on the one hand, by optimizing the set of calibration illuminants according to the sensor’s spectral response; and on the other hand, by developing strategies to prevent anomalous values in the weighting coefficients.

### 6.1. Optimization of the Set of Calibration Illuminants

As described in the previous sections, the procedure for optimizing the set of calibration illuminants for an LRS-PACF sensor using the MLR method would be as follows:

- Number of illuminants equal to or greater than the number of channels. If the number of illuminants equals the number of channels and any sensor reading can be expressed as a linear combination of the others, then (18) will not admit a valid mathematical solution.
- As illustrated in Section 3 with the two-channel fitting example shown in Figure 7, it is advisable to use predominantly quasi-monochromatic illuminants with spectral power distributions narrower than the sensitivity curves of the sensor channels, and approximately uniformly distributed across the sensor’s spectral range. This approach helps to balance the contribution of the squared error across the entire range and reduce multicollinearity when applying the MLR method.
- The illuminance levels used during calibration must be sufficient to ensure that sensor readings are significant with respect to the full-scale measurement range.
- It is especially important that, within the wavelength range where the target function $S_{\mathrm{mel}}\left(\lambda \right)$ is nonzero, the number of calibration illuminants be noticeably greater than the number of channels.
- Spectral regions where any sensor’s channel is sensitive must not be left without a significant energy contribution from at least one illuminant. This is the reason why a halogen lamp was included among the calibration sources in the previous examples, although the same effect could be achieved using infrared LEDs.
- Assuming that both the sensor’s spectral response and the LED-based illuminants’ emission spectra are approximately Gaussian, it is important to ensure that, across the sensor’s effective spectral range, the number of illuminant spectra exceeds the number of sensor response bells. Furthermore, the illuminants should be sufficiently narrowband to avoid producing similar outputs in adjacent channels.

**Figure 13:**
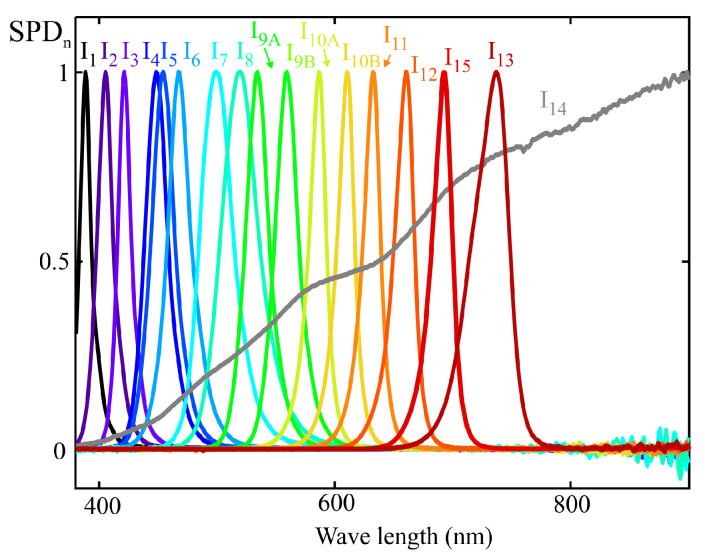
Illuminants’ normalized spectral distribution to improve calibration in the range 530–780 nm.

**Figure 14:**
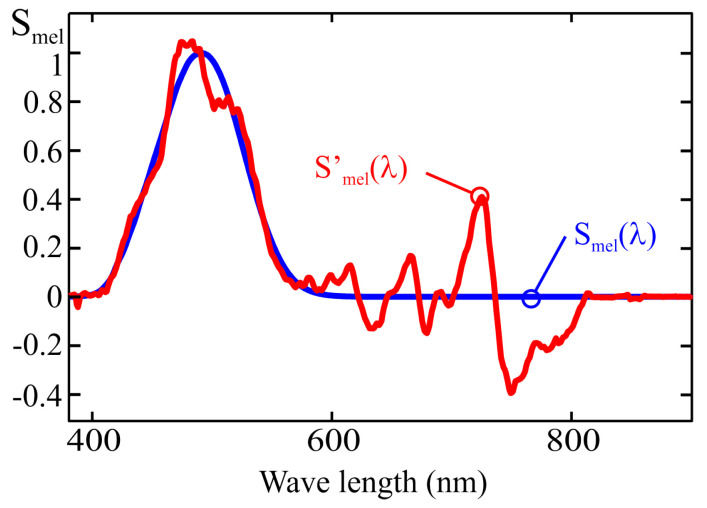
Improved spectral response of the sensor using 17 illuminants.

The latter rule is not fulfilled in the range between 530 and 780 nm in the example from Section 5, where the most significant deviations from the target spectral response are observed. In this region, the number of illuminant curves matches the number of sensor channels. A strategy to improve the fitting would therefore be to add an additional illuminant peaking within this range and avoid using non-monochromatic illuminants such as I_9_ and I_10_. Figure 13 shows the spectral distributions of the improved illuminant set in the 530–780 nm region. Illuminants I_9_ and I_10_ from Figure 9 have been replaced by illuminants I_9A_, I_9B_, I_10A_, and I_10B_, which are significantly more monochromatic. In addition, illuminant I_15_, peaking at 689 nm, has been included. The fitting obtained using the MLR method for this set of illuminants is shown in Figure 14. Compared to the fitting presented in Figure 12, it exhibits a lower deviation from the target curve, $S_{\mathrm{mel}}\left(\lambda \right)$. Nevertheless, the effects of multicollinearity between adjacent channels are still noticeable, leading to significant deviations in the 600–800 nm range. This error could be further reduced by increasing the number of illuminants; however, doing so would extend both the duration and complexity of the calibration process of each sensor, which is one of the main advantages of the MLR method used. Adding to this is the difficulty of finding commercial LEDs that meet these conditions. 

### 6.2. Identification of Anomalous Weighting Coefficients

As discussed in the previous section, for sensors where cross-channel interference is relatively low, it is expected that the weighting coefficients of the main channels (those whose peak sensitivity occurs at wavelengths close to the maximum of the target curve) will have a significantly higher relative weight compared to the others. Failure to meet this condition results in substantial deviations of the sensor’s spectral response from the target curve, as observed in Figure 12.

This provides a means to detect when an MLR-based fitting yields significant errors in the spectral response by comparing the weighting coefficients of the main channels with those of the secondary ones. When any secondary channel exhibits a weighting coefficient (either positive or negative) whose magnitude is comparable to or greater than that of the main channels, a substantial deviation from the target curve is to be expected. In such cases, as will be explained in subsequent sections, excluding one or more of these channels can help prevent the emergence of anomalous coefficients and improve the equivalent spectral response of the sensor.

**Figure 15:**
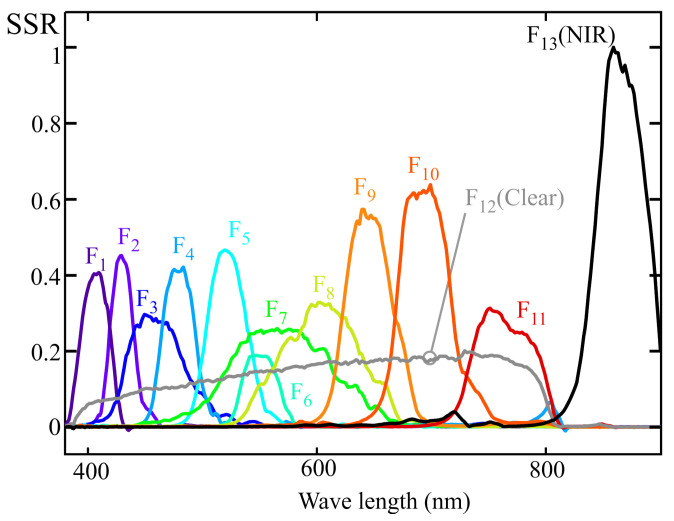
Experimental absolute spectral response of the 13-channel sensor.

However, for such a comparison of coefficients to be meaningful, the spectral responses of the sensor channels must be normalized. Figure 15 illustrates the variability in peak spectral sensitivity among the channels of the sensor characterized in Section 5. In this figure, the channel responses have been normalized with respect to the maximum value of channel F_13_ (NIR). It can be observed that channel F_13_ exhibits a peak sensitivity 5.3 times higher than that of channel F_6_. Moreover, for two sensors of the same model, the manufacturer reports up to a 66% variation in the same channel gain. These factors make prior normalization necessary in order to compare the weighting coefficients provided by the MLR fitting.

If the full spectral response of each channel is available, normalization is straightforward. However, obtaining such data requires a large number of measurements, as discussed in Section 2, thereby undermining one of the main advantages of the MLR method compared to the CIE E illuminant with a constant output of 1 mw/(nm · m^2^) used as a reference. Therefore, this study proposes an approximate normalization approach based on the same set of illuminants used for the MLR procedure.

Within the datasheets provided by the manufacturers for the sensors analyzed in this study, it can be observed that, although the peak sensitivity wavelength and the channel gain may vary significantly, the width of the spectral response curve remains approximately constant across sensors of the same model.

For a Gaussian distribution, the area under the curve meets the following relation:

(22) $$\begin{array}{c} A=\frac{1}{2}\cdot \sqrt{\frac{\pi }{\ln(2)}}\cdot Max\cdot FWHM, \end{array}$$

where FWHM stands for full width at half maximum (Max). The nominal value of this parameter can be found in the sensor datasheet. Assuming that the channel response resembles a Gaussian distribution and neglecting the area contribution from cross-sensitivity, the peak value of the spectral response for channel ‘n’ can be estimated using Expression (23), provided that a constant-power illuminant across all wavelengths of interest is available (i.e., the ideal CIE illuminant E).

(23) $$\begin{array}{c} Max_{n}=k\cdot \frac{Ch_{n}}{FWHM_{n}}, \end{array}$$

where $Ch_{n}$ stands for the reading of channel ‘n’ under the CIE E illuminant, and ‘k’ is a constant factor.

**Figure 16:**
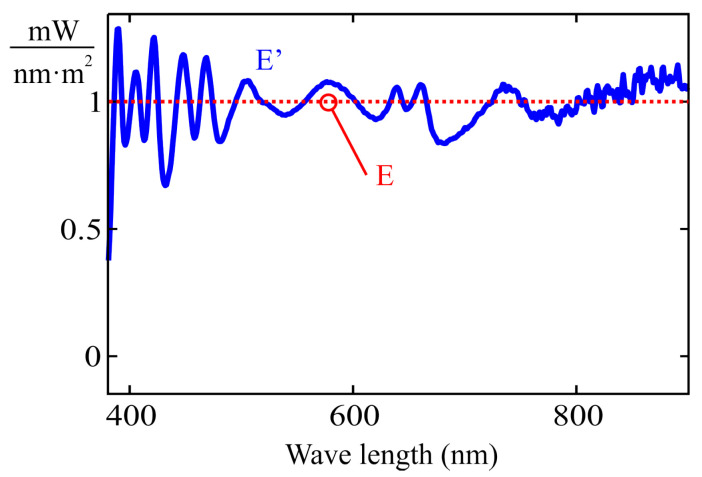
Quasi-constant spectral power distribution.

**Figure 17:**
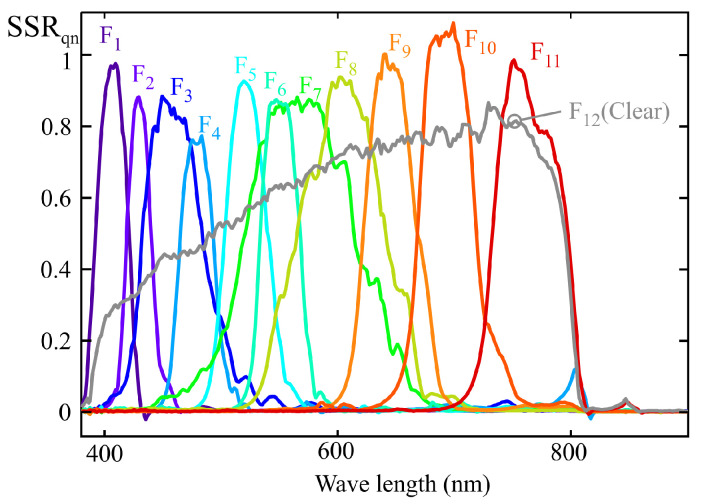
Quasi-normalized spectral sensitivity response.

The use of the CIE E illuminant would allow eliminating the effect caused by significant variations in the peak sensitivity wavelength of a channel. This ideal illuminant can be approximated by a weighted summation of the calibration illuminants used in the examples from Section 4 and Section 5 (see Figure 9). Figure 16 shows the approximation that minimizes the squared error, obtained using the MLR method. The spectral response of Figure 17 is obtained through the proposed approximate normalization method. The curve of each channel ‘n’ has been determined by multiplying the unnormalized response from Figure 15 by a scaling factor given by:

(24) $$\begin{array}{c} FE_{n}=\frac{FWHM_{n}}{Ch_{n}}. \end{array}$$

It becomes evident that, although the approximations do not provide exact normalization, the peaks of each channel yield comparable values that are closer to each other than those of the unnormalized response shown in Figure 15.

Based on this quasi-normalization, it is possible to identify when one of the weighting coefficients, obtained from the approximation of the melanopic sensitivity curve using the MLR method, exhibits a clearly anomalous value. As will be discussed in the following section, in such cases, eliminating one or more sensor channels may help mitigate the pendulum effect and reduce deviations in the spectral response.

### 6.3. Calibration Procedure via Selective Channel Elimination

**Figure 18:**
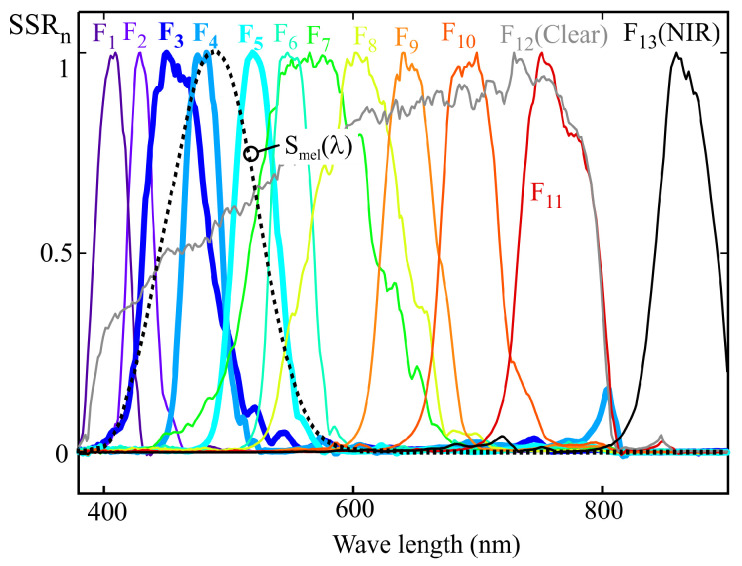
Normalized spectral sensitivity response of a AS7343 sensor obtained experimentally and objective sensibility curve $S_{\mathrm{mel}}\left(\lambda \right)$. Main channels F_3_, F_4_, and F_5_ are shown in bold.

As indicated in the previous sections, the main cause of the deviations observed in the spectral fitting obtained through the MLR method is the multicollinearity present in the data used for sensor calibration. Therefore, once the quasi-normalization described earlier has been applied, it becomes feasible to employ one of the optimized regression techniques aimed at mitigating the effects of multicollinearity that can be found in the literature, such as channel weighting regularization, variable selection, or ridge regression [24,25]. However, these methods are considerably more complex than MLR. For this reason, an alternative approach is proposed below, which leverages two specific characteristics of LRS-PACF sensor fitting using predominantly monochromatic illuminants to obtain target curves such as $S_{\mathrm{mel}}\left(\lambda \right)$ presented in this work:

- Based on the target curve and the approximate response curves provided by the manufacturer, it is possible to identify the channels that should dominate to achieve an accurate fit to the target spectral response. These channels are referred to as main channels in this paper. As shown in Figure 18 for the 13-channel sensor example, these correspond to F_3_, F_4_, and F_5_. The remaining channels, referred to as secondary channels, should have significantly lower weights in the model, as their spectral response exhibits substantial values in regions where the target response is low or null.
- When predominantly monochromatic illuminants are used, multicollinearity issues mainly arise between adjacent channels.

The proposed method consists of applying MLR, starting with the main channels and then sequentially incorporating secondary channels, removing from the model those that introduce coefficients exceeding a certain percentage of the highest coefficient among the main channels. This procedure is referred to as Selective Channel Elimination (SCE).

However, eliminating channels when applying the MLR method increases the total quadratic error for the calibration illuminants. As it will be discussed later, this can, in turn, improve the equivalent spectral response obtained and reduce the error when measuring illuminants that do not correspond to linear combinations of those included in the calibration set. Therefore, it is advisable to keep the number of discarded channels as low as possible.

As previously mentioned, the weighting coefficients associated with the main channels are the most critical for achieving an accurate fit to the sensor’s spectral response. Secondary channels primarily serve to compensate for the sensitivity exhibited by the main channels at wavelengths outside the main peak of their spectral response. Consequently, the absolute values of the weighting coefficients for these secondary channels should be significantly lower than those of the primary ones. The largest deviations in the calibrated spectral response obtained via the MLR method are typically caused by anomalous coefficients that violate this condition. Excluding the channels that produce such anomalous coefficients leads to a substantial reduction in the spectral response deviations observed in earlier sections (see Figure 10 and Figure 12). However, the order in which secondary channels are incorporated influences which ones are excluded and, therefore, affects the resulting spectral response. As mentioned earlier, the most significant deviations in the spectral response occur in regions where the number of illuminants is relatively low compared to the number of sensor response peaks, and these illuminants are not sufficiently monochromatic to prevent comparable readings between adjacent channels. Based on these considerations, the proposed order for incorporating channels into the previously described 13-channel sensor is as follows:

1. The process begins with the inclusion of the main channels. Main channels should provide the dominant coefficients of the model. They can be identified by comparing their approximate spectral response provided by the manufacturer with the target curve. For the proposed example, these correspond to channels F_3_, F_4_, and F_5_ (see Figure 18).
2. Since anomalous coefficients mainly arise between adjacent channels, the secondary channels are introduced by selecting the one that is furthest from those already tested. The distance between channels could be estimated from the nominal peak response wavelengths provided by the manufacturer. This ordering aims to minimize the number of discarded channels while reducing the error caused by the sensitivity of the primary channels in regions where the target curve exhibits very low or zero values. Based on this criterion, the recommended order for integrating the secondary channels is as follows: F_11_, F_9_, F_10_, F_1_, F_8_, F_6_, F_2_, and F_7_.
3. Finally, channel F_12_ (CLEAR) is incorporated. Although it does not exhibit a monochromatic spectral response, it demonstrates slightly higher sensitivity in the red region, where the primary channels tend to show increased spectral crosstalk, thus potentially contributing to a partial compensation of this effect.

**Figure 19:**
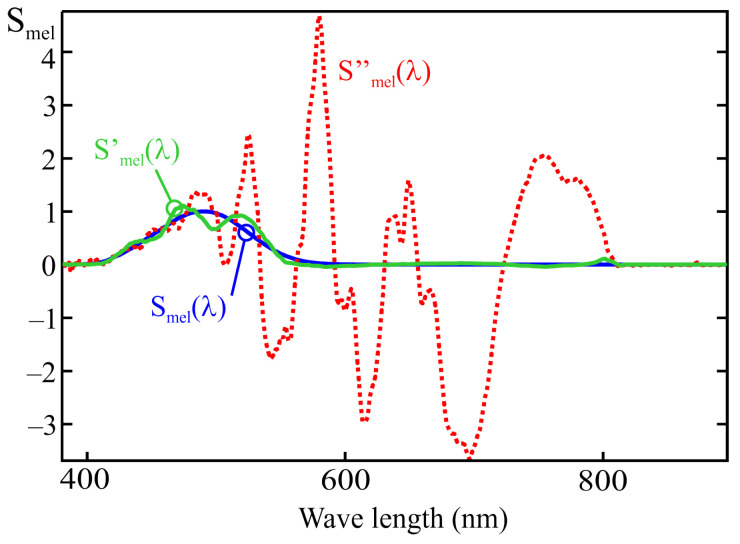
Comparison of the spectral response using selective channel elimination (SCE) and MLR.

Figure 19 shows the fit obtained using the same sensor as in Section 5, following the aforementioned order and applying a discard threshold for secondary coefficients equal to 25% of the maximum value of the main coefficients. In this curve (shown as $S_{\mathrm{mel}}^{\prime }\left(\lambda \right)$), channels F_2_, F_6_, and F_12_ have been discarded. A deviation with respect to the target curve $S_{\mathrm{mel}}\left(\lambda \right)$ significantly smaller than that obtained with the MLR method can be observed ($S_{\mathrm{mel}}^{\prime \prime }\left(\lambda \right)$).

## 7. Comparison of the Experimental Results Obtained Using the SCE and MLR Methods

**Figure 20:**
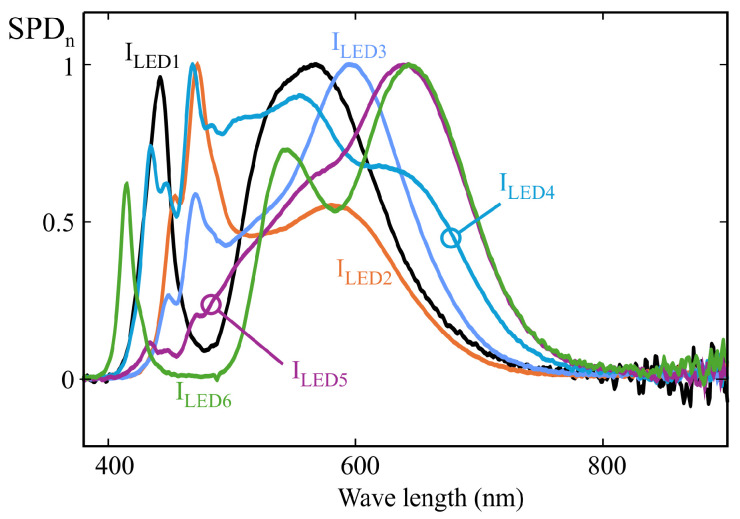
Normalized spectral power distribution of the white LEDs used as test illuminants.

Validation of the proposed methodology comprised the characterization of 10 different AS7343 sensors using both the MLR and SCE procedures. The calibration illuminants described in Section 4 and listed in Table 1 were employed for this purpose. Additionally, several white LEDs were used to compare the fitting obtained by both methods. Among these LEDs, several references specifically designed for integrative or circadian lighting systems were selected, including LEDs with either cyan boost and suppression features to activate or deactivate melatonin suppression mechanisms [4]. The normalized spectra of these LEDs are shown in Figure 20.

**Figure 21:**
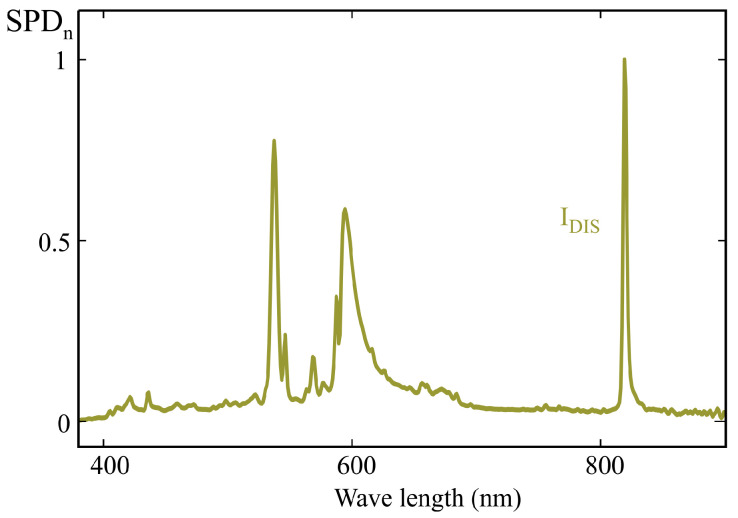
Normalized spectral power distribution of the 35W metal halide discharge lamp used as test illuminant.

Furthermore, although discharge lamps are currently being replaced by high-efficiency LEDs in virtually all applications, one such lamp was included among the test illuminants due to its strongly discontinuous spectrum, which makes it particularly sensitive to response deviations arising from the use of the MLR method, as discussed later in this section. The normalized spectrum of this lamp is shown in Figure 21. Table 2 details the references of all test illuminants used. 

Characterization was performed employing a 1.9 m Ulbricht sphere to which a 120 mm ancillary sphere featuring a symmetrical output port was attached. This ancillary sphere was 3D-printed and coated with a diffuse reflective paint based on barium sulfate. The sensors to be calibrated were mounted on it, along with an Ocean Optics (FL, US) HR2000+ spectrometer used as reference equipment. The reason for not using the splitter-based setup shown in Figure 5 was to allow the integration of low-cost diffuser lenses into the sensors, equivalent to those intended for the final application. The holographic diffuser lenses used in the system for cosine correction exhibit significantly lower attenuation across the entire measurement range, which was necessary to maximize signal levels at the monochromator output. However, their high cost makes them unsuitable for use in the target application. Because both the filters and the optical system configuration differ from those employed in Section 5, the spectral response of the sensor shown in Figure 15 does not exactly correspond to any of the sensors used in this section.

Using this setup, 10 AS7343 sensors were characterized by applying both the MLR and SCE methods. The same calibration illuminants as in the examples from Section 4 and Section 5 were used (see Figure 9). To compute the actual m-EDI values, the spectra recorded with the reference spectrometer were processed using the Python library LuxPy (Version 1.9.8) [5]. Table 3 summarizes the results, showing the calibration coefficients obtained with both methods after applying the quasi-normalization procedure described in Section 6.3. The full dataset is provided in the supplementary spreadsheets (Supplementary Materials).

The highest and lowest coefficients obtained by the MLR method are marked in red and blue, respectively, while the channels deactivated by the application of the SCE method are highlighted with an orange background. By examining the values highlighted in red and blue, it becomes evident that the pendulum effect is most pronounced in the coefficients corresponding to the adjacent channels F_6_ and F_7_. A significant spread between the maximum and minimum values obtained through the MLR method can be observed, which indicates that the resulting spectral response will exhibit substantial deviations from the target curve $S_{\mathrm{mel}}\left(\lambda \right)$.

Table 3 reveals substantial variability in the weighting coefficients obtained from different samples of the AS7343 sensor when applying the MLR and SCE methods. All samples were calibrated using the same set of illuminants, indicating that this variability arises from the inherent dispersion in the sensor’s characteristics. According to the manufacturer’s datasheet, the wavelength of peak sensitivity for each channel may deviate by 10 nm, and the gain may vary by up to 66% between sensors of the same model.

On the contrary, Table 4 presents the relative errors in m-EDI estimation obtained from the 10 analyzed samples for the test illuminants listed in Table 2. This table reveals a pronounced variability in the error obtained with the LED samples when applying the MLR calibration method, with errors ranging from 0.5% up to 885.7%, depending on the sensor and illuminant used. As expected, the error in m-EDI measurement for the metal halide lamp shown in Figure 21 reaches very high values, exceeding 1000% in one of the calibrated sensors.

When employing the selective channel elimination method, a markedly lower error is observed for virtually all the illuminants tested. The highest error recorded is 8.4% for sample S_7_ under illuminant I_LED1_; however, this value is substantially lower than the maximum 885.7% error obtained for this illuminant when applying the MLR method.

In addition to an appropriate selection of calibration illuminants, the proper operation of the SCE method requires a suitable choice of main channels and an appropriate adjustment of the discard threshold for secondary channels. The data presented in Table 5 allow these two aspects to be evaluated based on measurements from the 10 sensors mentioned above.

This table shows the RMS errors obtained by applying the MLR and SCE methods for both calibration illuminants and test LEDs at different discard threshold values. It can be observed that the RMS error for calibration illuminants is always lower when using the MLR method. With the SCE method, the error is higher, although it decreases as the threshold increases due to the reduced number of discarded channels (ndc). When the discard threshold is zero, the SCE method uses only the main channels, discarding the other nine. With this threshold, the table shows a lower RMS error for the test LEDs across all samples, with an average reduction from 17.63 to 1.77. This reduction would be less pronounced if the main channels were not correctly selected. Table 5 also shows that the RMS error for test LEDs using the SCE method decreases when the threshold increases from 0 to 10%, reaching its minimum for all samples at thresholds between 10% and 50% (values highlighted in green), and worsening at 60%. The lowest average RMS error across the 10 samples occurs for a threshold between 20% and 30%. Therefore, a 25% threshold was used to obtain the values in Table 3 and Table 4. Increasing the threshold from 0 to 25% reduces the RMS error for test LEDs from 1.77 to 1.36. Estimating the optimal threshold value depends on multiple factors that are difficult to quantify, such as the deviation of the adjusted illuminant E’ from illuminant E (see Figure 16), the sensitivity of primary channels at wavelengths outside their main peak, and variations in the nominal peak sensitivity wavelength of the sensor channels, among others. For this reason, an empirical adjustment based on the measurements shown in Table 5 was adopted in this work. Nevertheless, the table indicates that threshold values between 20% and 40% yield significant improvements compared to the MLR adjustment.

## 8. Conclusions

The m-EDI parameter is currently considered the most appropriate metric for quantifying the influence of lighting on human circadian rhythms. This paper focuses on one of the most affordable solutions currently available for measuring m-EDI: spectrometric sensors constructed using photodiode arrays combined with narrowband color filters. These sensors contain multiple channels, each of which is sensitive to a specific spectral region. In most commercially available models, each channel exhibits a spectral response that approximates a relatively narrow Gaussian curve. These sensors can be used to generate a spectral response suitable for m-EDI measurement by combining the responses of individual channels through weighted summation. However, sensor-to-sensor variations in channel responses require that these weighting coefficients be calculated individually for each sensor in order to ensure sufficient measurement accuracy.

The most commonly used calibration procedure consists of using a set of calibration illuminants and comparing the sensor outputs with the m-EDI values obtained using a reference sensor. Examples of this method, which is commonly based on multivariable linear regression to minimize the squared error over the selected calibration set, are frequently provided in manufacturer reference materials.

This paper analyzes the typical fitting errors associated with this calibration strategy and proposes improvements aimed at optimizing the spectral response for m-EDI estimation without significantly increasing the complexity of the calibration process. For this purpose, the spectral response of two sensors was characterized using a tunable monochromatic light source. These sensors contain nine and twelve channels, respectively. Due to the limited optical power of the light source, a method based on polynomial approximation was proposed to estimate the response of each channel in cases where the light source is not sufficiently monochromatic. This method compensates for the fact that the sensor response cannot be assumed to be constant across the wavelength range of the emitted light.

Based on the spectral response data obtained from these two sensors, the performance of the calibration method based on multivariable linear regression with a limited set of calibration illuminants was evaluated. The resulting equivalent spectral response was compared with the target melanopic sensitivity function $S_{\mathrm{mel}}\left(\lambda \right)$, revealing that significant discrepancies may arise. These deviations can result in large errors when measuring m-EDI under light sources whose spectral power distributions are not well represented by linear combinations of the selected calibration illuminants. A detailed examination of the equivalent spectral responses showed that the largest discrepancies are associated with the presence of anomalous weighting coefficients. These are defined as coefficients with absolute values considerably greater than those of the main channels, which are the channels whose peak sensitivity lies close to the maximum of the target melanopic function.

To reduce the deviation from the desired spectral response, two complementary strategies were investigated: optimization of the calibration illuminants and identification and exclusion of anomalous weighting coefficients. The first strategy generally involves increasing the number of calibration illuminants, which leads to a longer and more complex calibration procedure. The second strategy, involving the detection of anomalous coefficients, requires normalization of the channel responses to allow meaningful comparison between main and secondary channels. However, accurate normalization typically requires full knowledge of the spectral response of each channel, which is only obtainable through detailed characterization. This requirement contradicts the initial goal of maintaining a simple calibration process. To overcome this limitation, a method for approximate normalization based on the same set of calibration illuminants was proposed. This method uses the full width at half maximum of each channel response, a parameter generally available from the manufacturer and characterized by low sensor-to-sensor variability in the devices used during experimental validation.

A new procedure was then proposed to prevent the occurrence of anomalous coefficients by selectively excluding channels that introduce oscillatory deviations in the sensor’s spectral response. This phenomenon, referred to here as the pendulum effect, leads to distortions in the fitted spectral curve. The proposed solution is a modified version of the multivariable linear regression method in which the weighting coefficients of the main channels are calculated first. The remaining channels are then evaluated one at a time. If the inclusion of a given channel causes any of the previously calculated secondary coefficients to exceed a certain proportion of the highest main coefficient in absolute value, that channel is discarded by assigning it a zero coefficient. This approach produces spectral responses with significantly lower deviation compared to using all channels simultaneously in the regression.

The effectiveness of the proposed selective channel elimination procedure was validated experimentally using ten AS7343 sensors calibrated with a set of fourteen reference illuminants. The weighting coefficients obtained with this method were compared with those calculated using the standard multivariable regression approach. The corresponding m-EDI values were then computed for several test illuminants. The results demonstrated a significant reduction in measurement error when using the proposed method. For instance, the maximum error observed with the test LED illuminants reached 885.7% when using the conventional regression approach. This value was reduced to 8.4% when applying the selective elimination procedure.

## Figures and Tables

**Table 1:** Illuminants used for calibration.

Illuminant	Manufacturer	Part Number	Channel
Illuminant 1	Osram (DE)	LZ4-00UBH0-B1	
Illuminant 2	Osram (DE)	LZ4-00UBH0-B3	
Illuminant 3	Lumileds (NL)	LST1-01H07-VLT1-01	
Illuminant 4	Osram (DE)	LZ7-04M2PD	Blue
Illuminant 5	Osram (DE)	LZ4-40B208-0000	
Illuminant 6	Cree (US)	XPEBBL-L1-0000-00301-SB01	
Illuminant 7	Osram (DE)	LZ7-04M2PD	Cyan
Illuminant 8	Osram (DE)	LZ7-04M2PD	Green
Illuminant 9	Osram (DE)	LZ7-04M2PD	PC Lime
Illuminant 10	Osram (DE)	LZ7-04M2PD	PC Amber
Illuminant 11	Osram (DE)	LZ7-04M2PD	Red
Illuminant 12	Cree (US)	XQEEPR-00-0000000000A01-SB01	
Illuminant 13	Osram (DE)	LZ4-40R308-0000	
Illuminant 14	Lexman (FR)	2900 ºK Halogen lamp 115 W	

**Table 2:** Test illuminants.

Illuminant	Manufacturer	Type	Part Number	Power
I_LED1_	Osram, DE	Cool White 5500 K	LZ1-10CW02-0055	3.6 W
I_LED2_	Samsung, KR	Cyan enhanced white	LM302 LT-5902	8 W
I_LED3_	Samsung, KR	Cyan enhanced white	LM302 LT-5901	8 W
I_LED4_	Bridgelux, US	Cyan enhanced white	BXRV-TR-2750S-20A0-A-23	17 W
I_LED5_	Bridgelux, US	Warm white	BXRV-TR-2750S-20A0-A-23	17 W
I_LED6_	Soraa, US	Violet pump white LED	A60 Omni-11 W Zero Blue	11 W
I_DIS_	Philips, NL	Metal Halide	CDM-T 35W/830	35 W

**Table 3:** Sensor channel coefficients.

		F1	F2	F3	F4	F5	F6	F7	F8	F9	F10	F11	F12 (Clear)
SCE Intro Seq.		7	10	3	1	2	9	11	8	5	6	4	12
**S1**	**MLR coeff.**	4.470	3.013	5.208	−0.164	−17.456	53.848	−36.548	−30.913	14.304	8.131	12.692	−13.104
	**SCE coeff.**	−0.062	−0.010	0.212	0.723	0.663	0	0	−0.143	−0.057	−0.170	−0.159	0.161
**S2**	**MLR coeff.**	0.716	0.530	0.920	−0.106	−6.377	14.827	−10.504	−9.389	3.497	1.502	1.831	−1.968
	**SCE coeff.**	−0.016	0.060	0.217	0.834	0.831	0	0	−0.033	0.017	−0.045	−0.024	0
**S3**	**MLR coeff.**	−0.832	−0.486	−0.554	0.852	4.031	−9.379	6.588	5.282	−2.411	−1.691	−2.138	2.285
	**SCE coeff.**	−0.053	0.015	0.220	0.746	0.690	0	0	−0.111	−0.033	−0.174	−0.119	0.021
**S4**	**MLR coeff.**	0.015	0.049	0.235	0.391	−1.144	3.543	−2.869	−2.151	0.610	−0.073	0.073	−0.027
	**SCE coeff.**	−0.013	0.051	0.218	0.833	0.830	0	0	−0.035	0.017	−0.043	−0.025	0
**S5**	**MLR coeff.**	0.969	0.680	1.216	0.037	−6.592	15.872	−10.518	−9.960	4.250	2.191	2.459	−2.779
	**SCE coeff.**	−0.013	0.052	0.213	0.852	0.842	0	0	−0.041	0.025	−0.041	−0.028	0.013
**S6**	**MLR coeff.**	−0.802	−0.482	−0.573	0.631	2.969	−7.233	4.893	3.634	−2.071	−1.829	−2.115	2.345
	**SCE coeff.**	−0.049	0.008	0.234	0.742	0.702	0	0	−0.102	−0.038	−0.137	−0.113	0.011
**S7**	**MLR coeff.**	−0.730	−0.424	−0.466	0.695	2.957	−6.936	4.685	3.688	−1.969	−1.462	−1.917	2.024
	**SCE coeff.**	−0.043	0.014	0.209	0.736	0.706	0	0	−0.089	−0.121	−0.121	−0.121	0.019
**S8**	**MLR coeff.**	−0.865	−0.425	−0.747	0.606	2.712	−6.238	3.704	2.799	−1.244	−1.958	−2.348	2.554
	**SCE coeff.**	−0.014	0.066	0.209	0.857	0.828	0	0	−0.038	0.020	−0.047	−0.027	0.003
**S9**	**MLR coeff.**	−2.767	−1.510	−2.762	0.687	10.912	−26.243	17.013	−13.027	−7.510	−0.603	−7.163	8.035
	**SCE coeff.**	−0.014	0.058	0.193	0.850	0.842	0	0	−0.029	0.019	−0.043	0.013	0.140
**S10**	**MLR coeff.**	1.476	1.039	1.765	0.284	−8.562	24.996	−17.063	−16.144	5.503	2.642	4.278	−4.145
	**SCE coeff.**	−0.058	0.002	0.230	0.726	0.681	0	0	−0.124	−0.048	−0.154	0.140	0.140

**Table 4:** Percentual m-EDI error obtained with MLR and SCE for the test illuminants.

		White LED Lamp Samples						Discharge Lamp Sample
Sensor Sample		$I_{\mathrm{LED}1}$	$I_{\mathrm{LED}2}$	$I_{\mathrm{LED}3}$	$I_{\mathrm{LED}4}$	$I_{\mathrm{LED}5}$	$I_{\mathrm{LED}6}$	$I_{\mathrm{DIS}}$
**S1**	% **error MLR**	18.8%	30.7%	92.4%	84.0%	408.2%	885.7%	1229.6%
	% **error MLR**	7.5%	2.3%	2.4%	1.2%	1.6%	0.4%	0.6%
**S2**	% **error MLR**	0.5%	2.6%	2.4%	11.7%	66.3%	168.8%	325.2%
	% **error SCE**	4.6%	1.3%	3.9%	1.9%	2.8%	1.8%	5.1%
**S3**	% **error MLR**	12.3%	39.4%	29.3%	9.3%	51.7%	121.9%	182.7%
	% **error SCE**	8.0%	1.9%	2.4%	1.4%	2.9%	1.9%	2.4%
**S4**	% **error MLR**	2.4%	4.0%	4.5%	5.7%	19.1%	55.1%	71.0%
	% **error SCE**	5.9%	3.0%	3.1%	2.0%	3.2%	2.6%	5.9%
**S5**	% **error MLR**	2.6%	6.3%	18.4%	8.8%	55.3%	159.3%	334.1%
	% **error SCE**	6.5%	3.2%	4.1%	2.2%	3.5%	3.5%	5.7%
**S6**	% **error MLR**	12.0%	3.2%	10.1%	7.5%	40.6%	93.0%	136.1%
	% **error SCE**	7.0%	2.1%	2.5%	2.0%	2.8%	1.1%	2.7%
**S7**	% **error MLR**	11.0%	1.7%	8.9%	7.6%	44.6%	85.8%	131.2%
	% **error SCE**	8.4%	2.0%	2.2%	1.1%	2.1%	1.3%	2.8%
**S8**	% **error MLR**	9.3%	7.1%	6.6%	8.1%	30.8%	68.9%	117.6%
	% **error SCE**	7.9%	3.0%	2.0%	2.1%	2.9%	2.0%	6.1%
**S9**	% **error MLR**	11.7%	13.1%	32.8%	19.5%	121.6%	292.0%	520.0%
	% **error SCE**	3.4%	4.1%	2.9%	2.2%	2.5%	1.9%	4.3%
**S10**	% **error MLR**	2.0%	1.7%	37.5%	29.0%	159.5%	374.9%	547.9%
	% **error SCE**	6.9%	1.7%	1.9%	0.8%	1.4%	0.2%	2.7%

**Table 5:** RMS errors obtained by applying the MLR and SCE methods for both calibration illuminants and test LEDs at different discard threshold values.

		Threshold = 0%			Threshold = 10%			Threshold = 20%			Threshold = 30%			Threshold = 40%			Threshold = 50%			Threshold = 60%		
		rms Error Cal.	rms Error LED	ndc	rms Error Cal.	rms Error LED	ndc	rms Error Cal.	rms Error LED	ndc	rms Error Cal.	rms Error LED	ndc	rms Error Cal.	rms Error LED	ndc	rms Error Cal.	rms Error LED	ndc	rms Error Cal.	rms Error LED	ndc
**S1**	**MLR**	0.69	69.74	0	0.69	69.74	0	0.69	69.74	0	0.69	69.74	0	0.69	69.74	0	0.69	69.74	0	0.69	69.74	0
	**SCE**	2.51	1.50	9	1.81	1.44	3	1.81	1.44	3	1.79	1.19	2	1.28	1.81	2	1.28	1.81	2	1.28	1.81	2
**S2**	**MLR**	0.87	12.58	0	0.87	12.58	0	0.87	12.58	0	0.87	12.58	0	0.87	12.58	0	0.87	12.58	0	0.87	12.58	0
	**SCE**	3.70	2.03	9	3.08	1.69	3	3.08	1.69	3	3.08	1.69	3	3.08	1.69	3	2.08	2.50	2	2.08	2.50	2
**S3**	**MLR**	0.31	9.15	0	0.31	9.15	0	0.31	9.15	0	0.31	9.15	0	0.31	9.15	0	0.31	9.15	0	0.31	9.15	0
	**SCE**	2.51	1.43	9	1.86	1.09	3	1.85	1.01	2	1.30	1.47	2	1.34	1.37	2	1.34	1.37	2	1.34	1.37	2
**S4**	**MLR**	1.26	4.12	0	1.26	4.12	0	1.26	4.12	0	1.26	4.12	0	1.26	4.12	0	1.26	4.12	0	1.26	4.12	0
	**SCE**	3.67	2.05	9	3.07	1.61	3	3.07	1.61	3	3.07	1.61	3	3.07	1.61	3	3.07	1.61	3	2.07	2.38	2
**S5**	**MLR**	0.82	11.15	0	0.82	11.15	0	0.82	11.15	0	0.82	11.15	0	0.82	11.15	0	0.82	11.15	0	0.82	11.15	0
	**SCE**	3.83	2.27	9	3.18	1.70	3	3.18	1.70	3	3.18	1.70	3	3.18	1.70	3	3.18	1.70	3	2.15	2.49	2
**S6**	**MLR**	0.25	7.07	0	0.25	7.07	0	0.25	7.07	0	0.25	7.07	0	0.25	7.07	0	0.25	7.07	0	0.25	7.07	0
	**SCE**	2.58	1.51	9	1.90	1.19	3	1.89	1.07	2	1.89	1.07	2	1.35	1.53	2	1.35	1.53	2	1.35	1.53	2
**S7**	**MLR**	0.19	7.21	0	0.19	7.21	0	0.19	7.21	0	0.19	7.21	0	0.19	7.21	0	0.19	7.21	0	0.19	7.21	0
	**SCE**	2.54	1.49	9	1.93	1.12	3	1.93	1.04	2	1.93	1.04	2	1.37	1.57	2	1.37	1.57	2	1.37	1.57	2
**S8**	**MLR**	0.96	5.09	0	0.96	5.09	0	0.96	5.09	0	0.96	5.09	0	0.96	5.09	0	0.96	5.09	0	0.96	5.09	0
	**SCE**	3.54	2.16	9	2.93	1.48	3	2.93	1.48	3	2.93	1.48	3	2.93	1.48	3	2.00	2.04	2	2.00	2.04	2
**S9**	**MLR**	0.51	21.89	0	0.51	21.89	0	0.51	21.89	0	0.51	21.89	0	0.51	21.89	0	0.51	21.89	0	0.51	21.89	0
	**SCE**	3.38	1.82	9	2.77	1.52	3	2.77	1.52	3	2.77	1.52	3	2.77	1.52	3	1.86	2.03	2	1.86	2.03	2
**S10**	**MLR**	0.54	28.32	0	0.54	28.32	0	0.54	28.32	0	0.54	28.32	0	0.54	28.32	0	0.54	28.32	0	0.54	28.32	0
	**SCE**	2.55	1.48	9	1.81	1.07	3	1.81	1.07	3	1.79	0.87	2	1.26	1.47	2	1.26	1.47	2	0.97	0.91	1
**Average**	**MLR**	0.64	17.63	-	0.64	17.63	-	0.64	17.63	-	0.64	17.63	-	0.64	17.63	-	0.64	17.63	-	0.64	17.63	-
	**SCE**	3.08	1.77	-	2.43	1.39	-	2.43	1.36	-	2.37	1.36	-	2.16	1.57	-	1.88	1.76	-	1.65	1.86	-
**Standard deviation**	**MLR**	0.34	19.85	-	0.34	19.85	-	0.34	19.85	-	0.34	19.85	-	0.34	19.85	-	0.34	19.85	-	0.34	19.85	-
	**SCE**	0.58	0.33	-	0.61	0.25	-	0.62	0.28	-	0.70	0.30	-	0.90	0.13	-	0.73	0.34	-	0.43	0.53	-

(1) rms error cal.: rms error calibration illuminants. (2) rms error LED: rms error LED test illuminants. (3) ndc: number of discarded channels.

## Data Availability

This directory contains 10 spreadsheets with the calibration data of 10 AS7343 13-channel color sensors from AMS-Osram. Both methods (MLR and SCE) are applied in every sheet to obtain the coefficients that would allow the measurement of the m-EDI parameter through the weighted sum of the readings from each sensor’s channels.

## Supplementary Materials

The following supporting information can be downloaded at: https://zenodo.org/records/17307011 (accessed on 23 November 2025).

## Abbreviations

The following abbreviations are used in this manuscript:

CCD	Charge-Coupled Device
CIE	Commission Internationale de l’Éclairage/International Commission
	on Illumination
DUT	Device Under Test
FWHM	Full Width at Half Maximum
ipRGCs	Intrinsically Photosensitive Retinal Ganglion Cells
IR	Infrared
LED	Light-Emitting Diode
LRS-PACF	Low-resolution Spectrometric Sensors Built from Photodiode Arrays Combined
	with Narrowband Optical Filters
m-EDI	Melanopic Equivalent Daylight Illuminance
MLR	Multivariable Linear Regression
NIR	Near-infrared
RGB	Red, Green, Blue
SCE	Selective Channel Elimination
SPD	Spectral Power Distribution
SSRn	Normalized Spectral Sensitivity Response
UV	Ultraviolet

## References

[1] CIE CIE S 026/E:2018 CIE System for Metrology of Optical Radiation for ipRGC-Influenced Responses to Light. https://cie.co.at/publications/cie-system-metrology-optical-radiation-iprgc-influenced-responses-light-0.

[2] Zielinska-Dabkowska K.M., Schernhammer E.S., Hanifin J.P., Brainard G.C. (2023). Reducing nighttime light exposure in the urban environment to benefit human health and society. Science.

[3] Jägerbrand A.K., Spoelstra K. (2023). Effects of anthropogenic light on species and ecosystems. Science.

[4] Brown T.M., Brainard G.C., Cajochen C., Czeisler C.A., Hanifin J.P., Lockley S.W., Lucas R.J., Münch M., O’Hagan J.B., Peirson S.N. (2022). Recommendations for daytime, evening, and nighttime indoor light exposure to best support physiology, sleep, and wakefulness in healthy adults. PLoS Biol..

[5] Smet K.A.G. (2020). Tutorial: The LuxPy Python Toolbox for Lighting and Color Science. Leukos.

[6] (2021). Light and Lighting—Lighting of Work Places—Part 1: Indoor.

[7] Gilewski M. (2023). Selective Light Measurement in the Control of Reference LED Sources. Sensors.

[8] Gilewski M. (2023). Micro-Electro-Mechanical Systems in Light Stabilization. Sensors.

[9] Das A.J., Wahi A., Kothari I., Raskar R. (2016). Ultra-portable, wireless smartphone spectrometer for rapid, non-destructive testing of fruit ripeness. Sci. Rep..

[10] Trinh V.Q., Babilon S., Myland P., Khanh T.Q. (2022). Processing RGB Color Sensors for Measuring the Circadian Stimulus of Artificial and Daylight Light Sources. Appl. Sci..

[11] ams OSRAM Group (2020). ams OSRAM, AS7341—11-Channel Spectral Color Sensor Datasheet.

[12] ams OSRAM Group (2023). ams OSRAM, AS7343—14-Channel Spectral Color Sensor Datasheet.

[13] Espín F., Manzano E., Velásquez C., Chasi C., Andrade P., Vizuete M.Z., Botto-Tobar M., Casillas S., Gonzalez C., Sánchez C., Gomes G., Durakovic B. (2024). Modeling Variability in the Readings of an 8-channel Color Sensor and Its Uncertainty Estimation. Innovation and Research—Smart Technologies & Systems.

[14] Mohamed A., Kalavally V., Cain S.W., Phillips A.J.K., McGlashan E.M., Tan C.P. (2021). Wearable light spectral sensor optimized for measuring daily *α*-opic light exposure. Opt. Express.

[15] Shi N., Yang J., Cao Z., Jin X. (2024). A Programmable Ambient Light Sensor with Dark Current Compensation and Wide Dynamic Range. Sensors.

[16] Bäumker E., Zimmermann D., Schierle S., Woias P. (2021). A Novel Approach to Obtain PAR with a Multi-Channel Spectral Microsensor, Suitable for Sensor Node Integration. Sensors.

[17] Mohammadian N., Didikoglu A., Beach C., Wright P., Mouland J.W., Martial F.P., Johnson S., Van Tongeren M., Brown T.M., Lucas R.J. (2024). A Wrist-Worn Internet of Things Sensor Node for Wearable Equivalent Daylight Illuminance Monitoring. IEEE Internet Things J..

[18] Daschner R., Rothermel A., Rudorf R., Rudorf S., Stett A. (2018). Functionality and Performance of the Subretinal Implant Chip Alpha AMS. Sens. Mater..

[19] Wang W.W., Kwor R.Y., Longshore R.E. Crosstalk characterization in photodiode detector array using a 1-*μ*m optical scanning spot laser beam. Proceedings of the SPIE’s 1994 International Symposium on Optics, Imaging, and Instrumentation.

[20] Maham K., Vaskuri A., Manoocheri F., Ikonen E. (2020). Calibration of Near-Infrared Detectors Using a Wavelength Tunable Light Source. Opt. Rev..

[21] Lin R.L., Liu S.Y., Lee C.C., Chang Y.C. (2013). Taylor-Series-Expression-Based Equivalent Circuit Models of LED for Analysis of LED Driver System. IEEE Trans. Ind. Appl..

[22] Lin R.L., Tsai J.Y., Alonso J.M., Gacio D. (2015). Four-Parameter Taylor Series-Based Light-Emitting-Diode Model. IEEE J. Emerg. Sel. Top. Power Electron..

[23] McDowell R.J., Didikoglu A., Woelders T., Gatt M.J., Moffatt F., Notash S., Hut R.A., Brown T.M., Lucas R.J. (2024). Beyond Lux: Methods for species and photoreceptor-specific quantification of ambient light for mammals. BMC Biol..

[24] Akhtar N., Alharthi M.F., Khan M.S. (2024). Mitigating Multicollinearity in Regression: A Study on Improved Ridge Estimators. Mathematics.

[25] Efeizomor R.O. (2023). A Comparative Study of Methods of Remedying Multicolinearity. Am. J. Theor. Appl. Stat..

